# Integrated Transcriptomic Analysis and Functional Validation Identify CNTN1 as a Novel Metastatic Driver in Hilar Cholangiocarcinoma

**DOI:** 10.3390/biomedicines14030631

**Published:** 2026-03-11

**Authors:** Xiangming Ding, Chiyu Cai, Yuanxiang Lu, Zipeng Wang, Junjing Hou, Yushu Xue, Luyun Zhang, Meng Xie, Dongxiao Li

**Affiliations:** 1 Department of Gastroenterology, Henan Provincial People’s Hospital, People’s Hospital of Zhengzhou University, Zhengzhou 450003, China; dingxiangming@zzu.edu.cn (X.D.); xueyushu@gs.zzu.edu.cn (Y.X.); zhangluyun@gs.zzu.edu.cn (L.Z.); 2 Department of Hepatobiliary Surgery, Henan Provincial People’s Hospital, People’s Hospital of Zhengzhou University, Zhengzhou 450003, China; chiyu1101@gs.zzu.edu.cn (C.C.); wzpzzu@gs.zzu.edu.cn (Z.W.); junjing151729@gs.zzu.edu.cn (J.H.); 3 Department of Breast Surgery, Henan Provincial People’s Hospital, People’s Hospital of Zhengzhou University, Zhengzhou 450003, China; d202181970@hust.edu.cn

**Keywords:** hilar chlangiocarcinoma (HC), Contactin-1 (CNTN1), epithelial–mesenchymal transition (EMT), PI3K/AKT signaling, prognosis, metastasis

## Abstract

**Background:** Hilar cholangiocarcinoma (HC) is a highly aggressive malignancy with a poor prognosis, highlighting the urgent need to elucidate its molecular drivers. This study aimed to systematically identify and functionally validate key genes and pathways driving HC pathogenesis. **Methods:** RNA sequencing (RNA-seq) was performed on paired primary HC tumors and matched adjacent non-tumorous tissues to identify differentially expressed genes (DEGs). Subsequent bioinformatic analyses, including Gene Ontology (GO), Kyoto Encyclopedia of Genes and Genomes (KEGG) enrichment, and protein–protein interaction (PPI) network construction, were conducted to characterize the functional landscape and identify hub genes. Transwell assays and orthotopic metastatic models were used to investigate the functions of Contactin-1 (CNTN1) in HC invasion in vitro and metastasis in vivo. **Results:** RNA-seq analysis identified 35 DEGs in HC, mainly involved in cell adhesion, cytoskeletal regulation, and axon development. PPI network analysis identified six hub genes, including *CNTN1*, *NCAM1*, *PLP1*, *GPM6B*, *SLC1A3*, and *PMP2*. Furthermore, we demonstrated that CNTN1, a neuronal membrane glycoprotein, was markedly up-regulated in HC at both mRNA and protein levels, and its elevated expression correlated with poor prognosis. Gain- and loss-of-function studies demonstrated that *CNTN1* promotes HC cell invasion in vitro and metastasis in vivo. Mechanistically, *CNTN1* exerts its pro-invasive effects by activating the PI3K-AKT signaling pathway and inducing epithelial–mesenchymal transition (EMT). **Conclusions:** Our integrated analysis identifies *CNTN1* as a critical oncogenic driver in HC, promoting metastasis through PI3K-AKT-mediated EMT. These findings nominate *CNTN1* as a potential prognostic biomarker and therapeutic target in HC.

## 1. Introduction

Hilar cholangiocarcinoma (HC) is an aggressive malignancy originating from the extrahepatic bile ducts, accounting for approximately two-thirds of all biliary tract tumors and about 2% of solid malignancies [1,2]. Due to its complex anatomical location and highly metastatic propensity, the surgical resection rate remains unsatisfactory, often below 30% [3,4,5]. Even after curative surgery, patient outcomes remain poor, with overall survival rates of only 44.3% at 3 years, 32.5% at 5 years, and 19.9% at 10 years [6,7]. Frequent postoperative recurrence further contributes to this dismal prognosis, underscoring the pressing need to delineate the molecular mechanisms driving HC and to develop more effective treatment strategies.

Accumulating evidence indicates that the pathogenesis and progression of HC involve complex genetic and epigenetic alterations. Several molecules have emerged as potential prognostic markers and therapeutic targets. For instance, downregulation of *MTSS1* is associated with lymph node metastasis and poor differentiation in HC [8], while overexpression of *AQP1* correlates with tumor initiation, invasion, metastasis, and unfavorable prognosis [9]. Additionally, *PKM2* has been identified as a promising target implicated in HC neural invasion [10]. Despite these advances, the comprehensive molecular landscape and key regulatory networks underlying HC aggressiveness and metastasis remain incompletely understood.

Contactin-1 (CNTN1), a neuronal membrane glycoprotein known for its role in nervous system development [11,12], has recently gained attention in oncology. Aberrant *CNTN1* expression has been documented in several cancers, including prostate cancer, hepatocellular carcinoma, bladder cancer, and glioblastoma, where it is frequently associated with enhanced tumor progression and poor patient prognosis [13,14,15]. Functionally, CNTN1 has been characterized as an epithelial–mesenchymal transition (EMT)-associated protein that promotes cancer cell invasion, migration, and metastasis in various contexts [16,17]. Signaling pathways such as PI3K/AKT, VEGF-C/VEGFR-3, and MAPK have been reported to mediate its oncogenic effects. However, the expression pattern, functional significance, and clinical relevance of *CNTN1* in HC are still largely unexplored.

In this study, we aimed to systematically identify and validate key genes and pathways involved in HC pathogenesis. Using RNA sequencing (RNA-seq) of paired tumor and adjacent non-cancerous bile duct tissues (hereafter consistently referred to as adjacent non-tumor tissues), followed by integrative bioinformatics analysis, we identified *CNTN1* as a pivotal hub gene. Functional investigations demonstrated that *CNTN1* drives HC cell invasion and metastasis both in vitro and in vivo, mechanistically through activation of the PI3K-AKT pathway and induction of EMT. Our findings not only advance the molecular understanding of HC but also nominate *CNTN1* as a potential prognostic biomarker and therapeutic target for this lethal disease.

## 2. Materials and Methods

### 2.1. Patient Samples and Ethics Statement

This retrospective study was approved by the Ethics Committee of Henan Provincial People’s Hospital (Ethical Review (2025) No. 05) and conducted in accordance with the Declaration of Helsinki. Written informed consent was obtained from each participant prior to surgery. The study cohort consisted of 70 patients with histologically confirmed HC who underwent curative resection at Henan Provincial People’s Hospital (Zhengzhou, China) between 2014 and 2018. The inclusion criteria were: (1) pathologically confirmed primary HC; and (2) availability of complete clinicopathological and follow-up data. The exclusion criteria were as follows: (1) patients who received preoperative neoadjuvant chemotherapy or radiotherapy and (2) presence of other synchronous primary malignancies. The sample size of 70 represents a consecutive convenience cohort of all eligible cases meeting these criteria during the study period.

### 2.2. Specimens Processing and RNA-Seq

Patients undergoing radical resection for HC at the Department of Hepatobiliary Surgery, Henan Provincial People’s Hospital, were enrolled. All surgical specimens were confirmed by pathology as poorly differentiated adenocarcinoma of the hilar bile duct. Immediately after resection, paired HC tissue and adjacent non-cancerous bile duct tissue (obtained from the exact same patient, located at least 2 cm away from the tumor margin, and hereafter consistently referred to as adjacent non-tumor tissue) were immersed in RNAlater solution (Catalog No. AM7020, Thermo Fisher Scientific, Waltham, MA, USA) and stored at −80 °C until further processing.

Total RNA was extracted using TRIzol Reagent (Catalog No. 9108, TaKaRa, Kusatsu, Japan) following the manufacturer’s protocol. RNA quality and concentration were assessed with an RNA 6000 Nano Assay kit (Catalog No. 5067-1511, Agilent, Santa Clara, CA, USA). Sequencing libraries were constructed and sequenced on the BGISEQ-500 platform (BGI, Wuhan, China).

### 2.3. Cell Lines and Culture

The human extrahepatic cholangiocarcinoma cell lines EGI-1 and TFK-1 were obtained from the Henan Provincial Key Laboratory of Hepatobiliary and Pancreatic Diseases. Cells were cultured in Dulbecco’s Modified Eagle Medium (DMEM; Catalog No. 11995065, Gibco, Waltham, MA, USA) supplemented with 10% fetal bovine serum (FBS; Catalog No. 10099141, Gibco, USA) at 37 °C in a humidified atmosphere containing 5% CO_2_. Cell line identity was confirmed by short tandem repeat (STR) profiling, and routine *Mycoplasma* testing was performed using a commercial detection kit.

### 2.4. Migration and Invasion Assays

Cell migration and invasion were assessed using Transwell chambers with 8 µm pores (Catalog No. 4395, Corning, Corning, NY, USA). For invasion assays, the upper chamber was coated with 50 µL of Matrigel (diluted 1:8 in serum-free DMEM) and allowed to polymerize overnight. For migration assays, chambers were left uncoated. Cells were seeded in the upper chamber at densities of 5 × 10^4^ (migration) or 1 × 10^5^ (invasion) in serum-free medium, while the lower chamber contained complete medium as a chemoattractant. After incubation for 24 h (migration) or 72 h (invasion) at 37 °C with 5% CO_2_, cells that migrated or invaded to the lower surface were fixed, stained, and imaged. The number of cells was counted in five randomly selected fields per membrane, and each experiment was performed in triplicate.

### 2.5. Lentivirus Construction and Transfection

Short hairpin RNA (shRNA) targeting *CNTN1* (sh*CNTN1*) was cloned into the pLKO.1-puro vector (Genechem, Shanghai, China). For overexpression, the full-length *CNTN1* cDNA was cloned into a pLKO.1-puro lentiviral vector (DesignGene Biotechnology, Shanghai, China). Cells were transfected with lentiviral particles at a multiplicity of infection (MOI) of 10–30 for 12 h, followed by replacement with fresh DMEM containing 10% FBS. Knockdown or overexpression efficiency was validated 48–72 h post-transfection by quantitative real-time PCR (RT-qPCR) and Western blotting.

### 2.6. RNA Extraction and RT-qPCR

Total RNA from clinical specimens (extracted as described in Section 2.2) and transfected cells was reverse-transcribed into cDNA using the PrimeScript RT Reagent Kit (Catalog No. RR037A, TaKaRa, Kusatsu, Japan). qPCR was performed using SYBR Premix ExTaq (Catalog No. DRR420A, TaKaRa, Kusatsu, Japan) on an ABI StepOne system (Applied Biosystems, Foster City, CA, USA). The cycling conditions were: 40 cycles of 95 °C for 30 s, 60 °C for 5 s, and 70 °C for 30 s. Relative gene expression levels were quantified utilizing the 2^−ΔΔCt^ method with GAPDH as the endogenous control.

### 2.7. In Vivo Metastasis Assay and Bioluminescence Imaging

All experiments involving animals were approved by the experimental animal ethics committee of Henan Provincial People’s Hospital. All animal procedures were carried out in accordance with the Guide for the Care and Use of Laboratory Animals and standards articulated in the Animal Research: Reporting of In Vivo Experiments. A metastatic model was established in mice as previously described [18,19], with slight modifications. Briefly, 6 × 106 cells were suspended in PBS, mixed with Matrigel (Catalog No. 356234, BD Biosciences, San Jose, CA, USA), and injected orthotopically into the left liver lobes of BALB/c nude mice (male, 4-week-old). Each treatment group consisted of 10 mice. For in vivo monitoring, cells were infected with luciferase-expressing lentivirus (Lv-luc-blast, Hanbio, Shanghai, China) and selected with blasticidin (Catalog No. ant-bl-1, InvivoGen, Toulouse, France) for two weeks. D-Luciferin (Catalog No. LUCK-100, Gold Biotechnology, St. Louis, MO, USA) was injected weekly intraperitoneally into each mouse for monitoring tumor formation and metastasis, and images were captured with a Lago X optical imaging system (SI Imaging, Seattle, WA, USA). Lung tissues were dissected after 10 weeks, fixed with 4% paraformaldehyde, and stained with hematoxylin and eosin.

### 2.8. Statistical Analysis

Data are presented as mean ± standard error of the mean (SEM) from at least three independent experiments. Comparisons between two groups were performed using Student’s *t*-test (for parametric data) or the Mann–Whitney U test (for non-parametric data). Categorical variables were analyzed using Fisher’s exact test. Survival analysis was conducted using the Kaplan–Meier method, with differences assessed by the log-rank test. Univariate and multivariate analyses were performed using Cox proportional hazards models. All statistical analyses were conducted with SPSS software (version 19.0), and a two-sided *p*-value < 0.05 was considered statistically significant.

### 2.9. Supplementary Methods

Detailed protocols for additional experiments are provided in the Supplementary Materials.

## 3. Results

### 3.1. Identification of Differentially Expressed Genes in Hilar Cholangiocarcinoma

**Figure 1:**
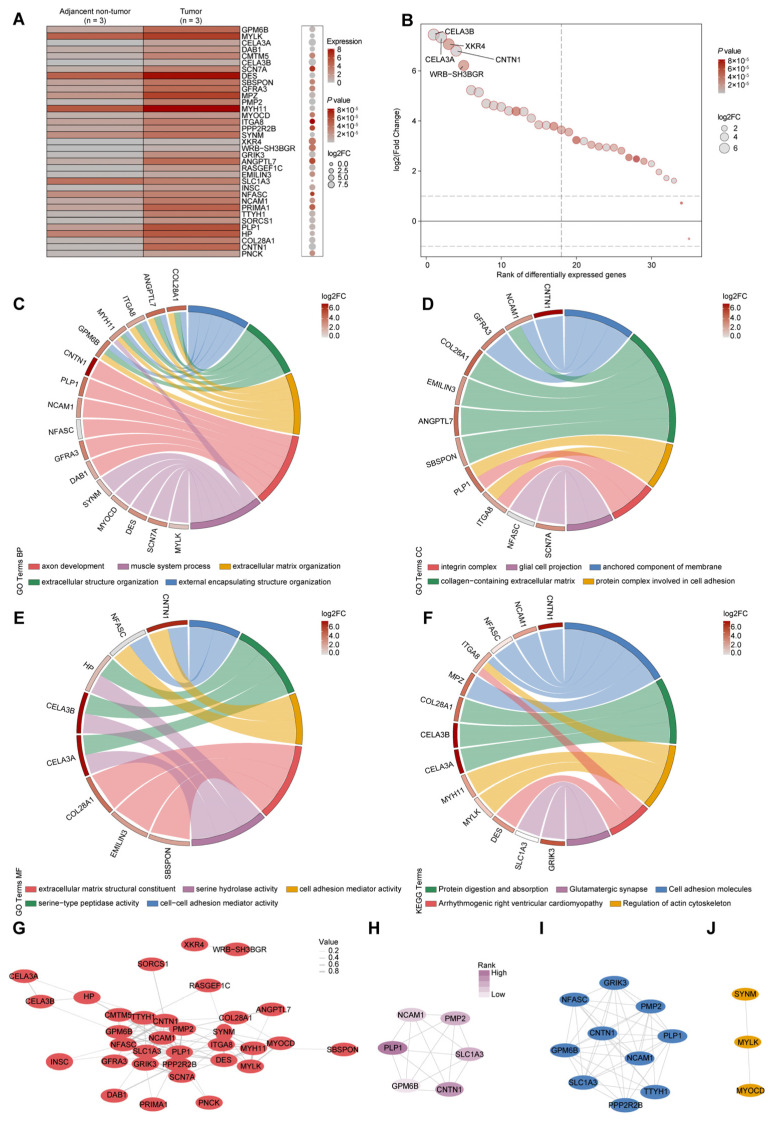
Identification and functional analysis of DEGs in hilar cholangiocarcinoma. (**A**) Heatmap depicting the expression patterns of 35 DEGs between HC and adjacent non-tumorous tissues. (**B**) Top five DEGs with the greatest log_2_ fold-change values. (**C**–**E**) GO enrichment analysis of DEGs in biological processes (**C**), cellular components (**D**), and molecular functions (**E**). (**F**) KEGG pathway enrichment analysis of DEGs. (**G**) PPI network constructed from DEGs using the STRING database and visualized with Cytoscape. (**H**) Identification of six hub genes with the highest degree of connectivity in the PPI network. (**I,J**) Two functional modules identified within the PPI network using the MCODE algorithm.

RNA sequencing analysis of 3 paired HC and adjacent non-tumorous tissues revealed 35 differentially expressed genes (DEGs). A heatmap illustrates the expression patterns of these DEGs (Figure 1A). Among them, 34 genes were up-regulated and one was down-regulated (Supplementary Table S1 and Supplementary Figure S1). The top five DEGs displaying the greatest log_2_ fold-changes were *CEL3B*, *CEL3A*, *XKR4*, *CNTN1*, and *WRB-SH3BGR* (Figure 1B).

### 3.2. Functional Enrichment Analyses of DEGs

To explore the biological relevance of the DEGs in HC, we performed Gene Ontology (GO) and Kyoto Encyclopedia of Genes and Genomes (KEGG) enrichment analyses. GO analysis revealed significant enrichment across three main categories. In biological processes (BP), the DEGs were associated with axon development, muscle system processes, extracellular matrix organization, extracellular structure organization, and external encapsulating structure organization (Figure 1C). For cellular components (CC), DEGs were enriched in the integrin complex, glial cell projection, anchored component of membrane, collagen-containing extracellular matrix, and protein complexes involved in cell adhesion (Figure 1D). Within molecular functions (MF), the DEGs functioned as extracellular matrix structural constituents and displayed serine hydrolase activity, cell adhesion mediator activity, serine-type peptidase activity, and cell–cell adhesion mediator activity (Figure 1E). KEGG pathway analysis further highlighted significant enrichment in pathways related to cell adhesion molecules (CAMs) and regulation of the actin cytoskeleton, both closely linked to tumor cell migration and invasion (Figure 1F). Additional enriched pathways included glutamatergic synapse, protein digestion and absorption, and arrhythmogenic right ventricular cardiomyopathy (Figure 1F). Together, these findings suggest that the identified DEGs are predominantly involved in cell adhesion and cytoskeletal dynamics, which may contribute to HC progression.

### 3.3. Protein–Protein Interaction Network and Hub Gene Identification

To delineate functional relationships among the DEGs, we constructed a protein–protein interaction (PPI) network using the STRING database and visualized it with Cytoscape (version 3.10) (Figure 1G). Degree-centrality analysis via the cytoHubba plugin identified the six most interconnected nodes as hub genes, including *CNTN1*, *NCAM1*, *PLP1*, *GPM6B*, *SLC1A3*, and *PMP2* (Figure 1H). Furthermore, the MCODE algorithm uncovered two densely connected functional modules within the network (Figure 1I,J). Collectively, the identification of central hubs and functional modules reveals a coordinated regulatory network that underlies HC progression.

### 3.4. Experimental Validation of Hub Gene Expression

We next examined the expression of the candidate hub genes by RT-qPCR in an expanded cohort of 70 paired primary HC and adjacent non-neoplastic tissues. Compared with non-tumorous tissues, HC samples showed significantly elevated mRNA levels of *CNTN1*, *NCAM1*, *PLP1*, *GPM6B*, and *PMP2*, whereas the expression of *SLC1A3* was markedly down-regulated (Supplementary Figure S2). These findings support the potential role of these hub genes in HC pathogenesis.

### 3.5. Increased CNTN1 Expression Predicts a Poor Prognosis in HC

**Figure 2:**
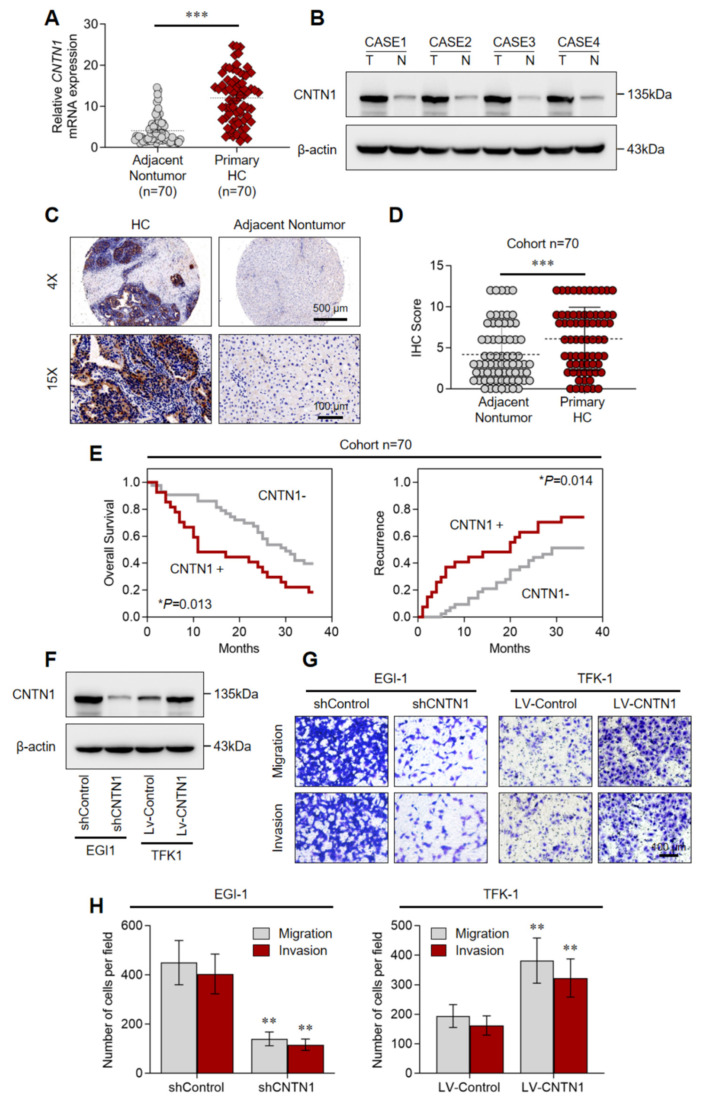
*CNTN1* predicts a poor prognosis in HC and facilitates HC cell migration and invasion in vitro. (**A**) Relative mRNA expression of *CNTN1* in primary HC tissues (*n* = 70) compared to adjacent nontumor tissues (*n* = 70). Data are presented as mean ± SEM. *** *p* < 0.001. (**B**) Representative Western blot showing CNTN1 protein levels in four paired HC (T) and adjacent nontumor (N) tissue samples. β-actin serves as a loading control. (**C**,**D**) Representative IHC images (C) and quantification of IHC scores (**D**) for CNTN1 protein expression in HC and adjacent nontumor tissues. Scale bars, 500 µm (upper), 100 µm (lower). *** *p* < 0.001. (**E**) Kaplan–Meier analysis was used to determine the correlation between *CNTN1* expression and recurrence or OS in HC cohort. * *p* < 0.05. (**F**) Western blot analysis of CNTN1 expression in the indicated cells. β-actin serves as a loading control. (**G**,**H**) Representative images (**G**) and quantitative analysis (**H**) of Transwell migration (upper panels) and invasion (lower panels) assays of the indicated HC cells. Scale bar: 100 µm. Data are presented as the mean number of cells per field ± SEM from three independent experiments. * *p* < 0.05, ** *p* < 0.01.

Emerging evidence indicates that elevated *CNTN1* expression correlates with cancer progression and unfavorable clinical outcomes [13,14]. We therefore investigated its clinical relevance in HC. RT-qPCR analysis of 70 paired primary HC and adjacent non-tumor samples confirmed that *CNTN1* mRNA levels were significantly higher in tumor tissues (Figure 2A). The increased *CNTN1* expression was further confirmed at the protein level by Western blot (Figure 2B). Consistent with these findings, IHC staining revealed pronounced up-regulation of *CNTN1* in HC specimens from an independent patient cohort (Figure 2C–D). Clinically, elevated *CNTN1* expression was associated with aggressive tumor behavior, including a higher TNM stage (Table 1). Kaplan–Meier survival analysis further demonstrated that patients with elevated *CNTN1* levels had significantly shorter overall survival and a greater risk of tumor recurrence (Figure 2E). Collectively, these results indicate that *CNTN1* is frequently up-regulated in HC and correlates with aggressive tumor behavior and poor prognosis, supporting its potential role as a prognostic biomarker in this malignancy.

### 3.6. CNTN1 Facilitates HC Invasion and Metastasis In Vitro and In Vivo

To investigate the functional role of *CNTN1* in HC progression, we performed gain- and loss-of-function experiments. The EGI-1 cholangiocarcinoma cell line, which exhibits high endogenous *CNTN1* expression, was used to establish a stable *CNTN1*-knockdown cell strain and was denoted as EGI-1-sh*CNTN1* (Figure 2F). Conversely, the TFK-1 cell line, which expresses low basal levels of *CNTN1*, was transduced with a *CNTN1*-overexpressing lentivirus (LV) and designated as TFK-1-LV-*CNTN1* (Figure 2F).

**Figure 3:**
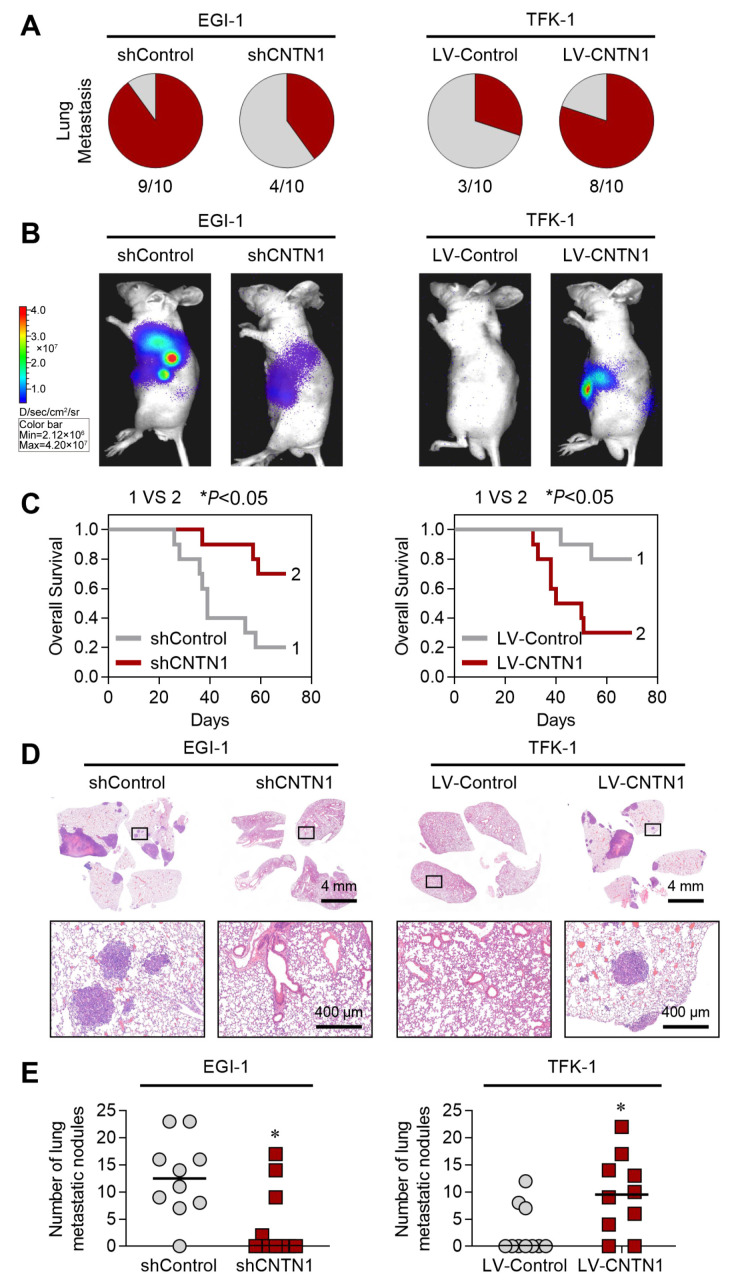
CNTN1 promotes HC metastasis in vivo. (**A**) Incidence of lung metastasis of each group after orthotopic xenografting with the indicated HC cells. (**B**) Representative bioluminescent images of the different groups. (**C**) The overall survival times of each group after orthotopic transplantation with the indicated cells. * *p* < 0.05. (**D**) Representative H&E staining of lung sections from each group, showing metastatic nodules. Scale bars: 4 mm (overview) and 400 µm (magnified view). (**E**) The number of metastatic lung nodules of each group. * *p* < 0.05.

Transwell assay showed that CNTN1 knockdown significantly impaired the migratory and invasive capabilities of EGI-1 cells (Figure 2G,H). In contrast, *CNTN1* overexpression markedly enhanced the migration and invasion of TFK-1 cells (Figure 2G,H). We further evaluated the metastatic role of *CNTN1* in vivo using an intrahepatic orthotopic xenograft model in nude mice. Down-regulation of *CNTN1* notably suppressed pulmonary metastasis and prolonged overall survival (Figure 3A–E). Conversely, *CNTN1* overexpression strongly promoted pulmonary metastasis and was associated with shorter survival (Figure 3A–E). Together, these results demonstrate that *CNTN1* facilitates HC invasion and metastasis both in vitro and in vivo, underscoring its functional contribution to tumor aggressiveness.

### 3.7. CNTN1 Promotes EMT and Invasion Through AKT Activation in HC Cells

**Figure 4:**
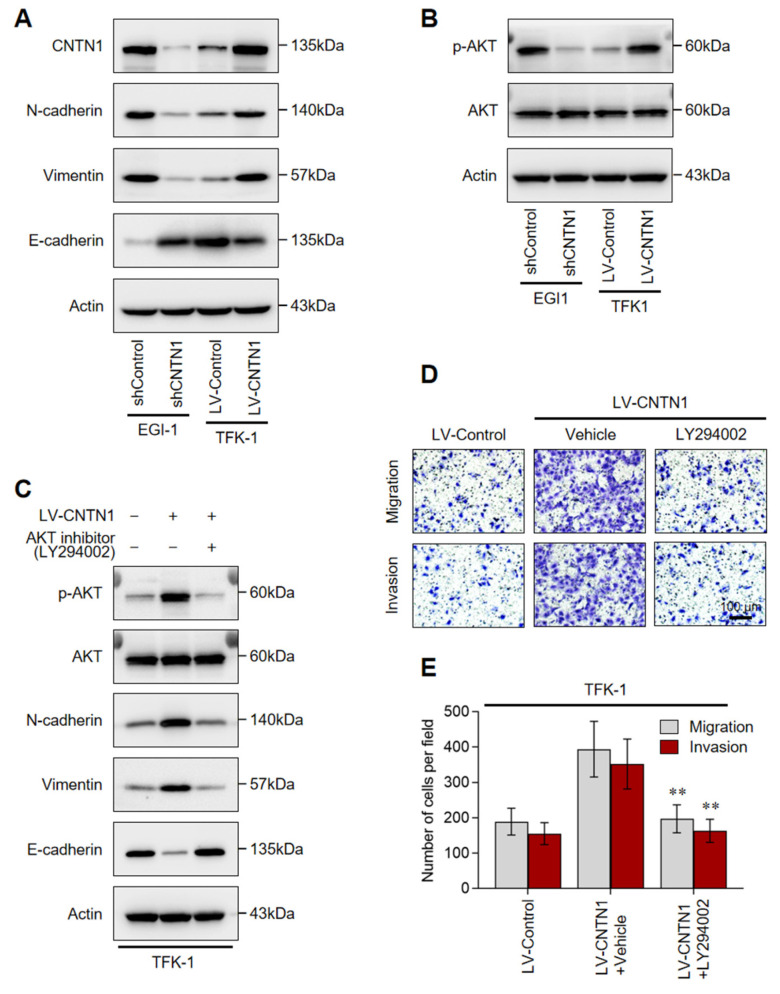
*CNTN1* activated PI3K-AKT signaling to promote EMT in HC cells. (**A**) Western blot analysis of CNTN1 and EMT markers (N-cadherin, Vimentin, and E-cadherin) in EGI-1 cells with *CNTN1* knockdown and TFK-1 cells with *CNTN1* overexpression. (**B**) Protein levels of total AKT and phosphorylated AKT in the indicated cell lines. (**C**) Western blot analysis of EMT markers, total AKT, and p-AKT in TFK-1-LV-*CNTN1* cells treated with the PI3K inhibitor LY294002 or vehicle control. (**D**,**E**) Transwell migration (upper panels) and invasion (lower panels) assays of TFK-1 LV-Control and LV-*CNTN1* cells treated with or without LY294002. Representative images are shown in (**D**), and quantitative analysis of migrating and invading cells is presented in (**E**). Scale bar: 100 µm. Data are mean ± SEM from three independent experiments. ** *p* < 0.01.

EMT is a critical process driving tumor cell invasion and metastasis [20,21]. As *CNTN1* has been implicated in EMT regulation [16,17], we aimed to determine whether it induces EMT in HC cells. Western blotting revealed that knockdown of CNTN1 in EGI-1 cells significantly reduced the expression of mesenchymal markers N-cadherin and Vimentin, while increasing the epithelial marker E-cadherin (Figure 4A). Conversely, *CNTN1* overexpression in TFK-1 cells elevated N-cadherin and Vimentin levels and suppressed E-cadherin expression (Figure 4A).

The PI3K-AKT pathway is one of the major pathways involved in tumor progression and the EMT phenomenon [20,21]. We therefore investigated whether *CNTN1* promotes EMT in HC cells by activating the PI3K-AKT pathway. Western blot demonstrated that CNTN1 overexpression enhanced AKT phosphorylation, whereas *CNTN1* knockdown reduced AKT phosphorylation (Figure 4B). To determine whether PI3K-AKT signaling mediates *CNTN1*-induced EMT, we treated *CNTN1*-overexpressing TFK-1 cells with the PI3K inhibitor LY294002. Inhibition of PI3K markedly attenuated the *CNTN1*-driven EMT phenotype, as evidenced by restored E-cadherin and decreased N-cadherin/Vimentin expression (Figure 4C). Consistently, the enhanced migratory and invasive capacities of *CNTN1*-overexpressing cells were significantly suppressed by LY294002 treatment (Figure 4D,E). These results demonstrated that *CNTN1* promotes EMT and invasion through activation of the PI3K-AKT signaling pathway (Supplementary Figure S3).

## 4. Discussion

HC remains a significant clinical challenge due to its aggressive behavior, characterized by early local invasion, frequent metastasis, and high rates of recurrence even after curative resection [1,2,3]. This poor prognosis highlights the urgent need to define the molecular drivers of HC progression. In the present study, by integrating RNA sequencing, bioinformatic analyses, and functional assays, we identified a DEG signature associated with invasive traits in HC, enriched in pathways related to cell adhesion and cytoskeletal regulation. *CNTN1* emerged as a central hub gene within this signature. Further investigations revealed that *CNTN1* is not only upregulated in clinical HC samples and correlates with adverse clinical outcomes, but also functionally promotes HC cell invasion and metastasis by inducing EMT through activation of the PI3K/AKT signaling pathway.

PPI and functional module analyses revealed six hub genes linked to oncogenesis and progression in HC. Among these, CNTN1, a neuronal membrane glycoprotein, has gained increasing attention for its oncogenic role in various malignancies. Interestingly, while normally restricted to neural tissues, the ectopic expression of *CNTN1* has recently been identified as a potent driver in several adenocarcinoma subtypes. Mechanistic studies in prostate and lung adenocarcinomas reveal that *CNTN1* does not merely enhance migration, but fundamentally reprograms tumor cells by actively driving epithelial–mesenchymal plasticity and conferring chemoresistance [13,14,22]. This “neural-to-mesenchymal” phenotypic shift mediated by *CNTN1* is strongly associated with advanced tumor stages, lymph node metastasis, and poor survival in these glandular malignancies. Given that HC is an aggressive adenocarcinoma characterized by frequent perineural invasion and high metastatic propensity, our findings parallel its established pro-metastatic roles in other adenocarcinomas. Specifically, we demonstrated that *CNTN1* overexpression promotes HC cell migration, invasion, and intrahepatic metastasis in vivo, whereas *CNTN1* knockdown markedly suppresses these malignant behaviors. Clinically, elevated *CNTN1* expression in primary HC tumors correlates with worse overall survival and higher recurrence rates, supporting its potential utility as a prognostic biomarker.

EMT is a pivotal mechanism that drives tumor cell invasion, migration, and metastasis [20,21]. *CNTN1* has been implicated in promoting EMT in other cancers, a process typically marked by decreased E-cadherin and increased N-cadherin and Vimentin expression [16,17]. In HC, our gain- and loss-of-function experiments demonstrated that *CNTN1* knockdown reversed the EMT phenotype and impaired invasiveness, whereas its overexpression elicited the opposite effects. The profound inverse relationship observed between the neural adhesion molecule *CNTN1* and the canonical epithelial adhesion molecule E-cadherin warrants particular mechanistic attention. During HC progression, the ectopic accumulation of *CNTN1* appears to actively dismantle epithelial integrity rather than merely correlating with its loss. As a GPI-anchored neuronal glycoprotein, aberrant CNTN1 expression at the cell surface may initiate an “adhesion molecule reprogramming” that overrides the default E-cadherin-mediated adherens junctions. Our data strongly suggest that this cadherin switch is executed intracellularly via the PI3K/AKT signaling axis. It is well-documented that hyperactive AKT signaling cascades phosphorylate and inactivate GSK-3β, thereby preventing the degradation of master EMT transcription factors such as Snail. The stabilized Snail subsequently translocates to the nucleus and directly represses the transcription of the E-cadherin gene (*CDH1*) [23,24]. Thus, *CNTN1* does not just passively mark EMT; it acts as an upstream orchestrator that extinguishes the epithelial adhesion program to facilitate tumor dissemination.

Notably, the pro-EMT and pro-invasive effects of *CNTN1* were abolished by the PI3K inhibitor LY294002, confirming the PI3K/AKT pathway as a key downstream effector of *CNTN1* signaling in HC. Given that several PI3K/AKT inhibitors are under clinical investigation [25,26], our findings suggest that patients with *CNTN1*-high HC tumors may particularly benefit from pharmacological blockade of this downstream pathway. Furthermore, considering its specific localization on the extracellular membrane, *CNTN1* emerges as a highly attractive candidate for direct intervention. The development of *CNTN1*-targeting neutralizing monoclonal antibodies, potentially in combination with these downstream PI3K/AKT pathway inhibitors, could emerge as a promising precision medicine strategy to block tumor spread and improve survival in patients with advanced HC.

Intriguingly, our bioinformatic analysis also highlighted other DEGs, including *PLP1*, *NCAM1*, *NFASC*, *GFRA3*, and *DAB1*, which are predominantly associated with nervous system structure and function [27,28,29,30,31]. This finding takes on particular significance in the context of HC, a malignancy well-documented for its propensity for perineural invasion (PNI), a key route for local spread, and a negative prognostic factor [32,33,34]. The recurrent emergence of neural-related molecules in our analysis suggests a broader thematic link between neural pathobiology and HC aggressiveness. As a canonical neural adhesion molecule, *CNTN1* may serve as a molecular mediator that facilitates tumor–nerve interactions and thereby promotes local invasion and metastatic niche formation, suggesting a valuable direction for further investigation of the neurotropic nature of HC.

Despite these promising findings, several limitations of the present study must be explicitly acknowledged. First, our clinical validation was based on a retrospective cohort from a single center. Future large-scale, multicenter prospective studies are necessary to fully establish the clinical utility of CNTN1 as an independent prognostic biomarker. Second, while we elucidated the downstream PI3K/AKT signaling axis, the precise extracellular interactome and the specific structural domains through which this GPI-anchored protein initiates tumor-nerve cross-talk remain incompletely defined. Future investigations focusing on the structural biology of *CNTN1* within the tumor microenvironment are warranted to facilitate its clinical translation.

## 5. Conclusions

In summary, our integrated approach combining RNA sequencing, bioinformatic analysis, and functional validation identifies CNTN1 as a critical hub gene in HC progression. We demonstrate that *CNTN1* promotes HC cell invasion and metastasis, mechanistically through activation of the PI3K-AKT pathway and induction of EMT. Collectively, these findings provide mechanistic insight into HC progression and nominate *CNTN1* as a candidate biomarker and therapeutic target for this lethal malignancy.

## Figures and Tables

**Table 1:** Correlation between *CNTN1* expression and clinicopathological characteristics in a cohort of patients with hilar cholangiocarcinoma.

Clinicopathological Variables		*CNTN1* Expression, *n* (%)		*p*-Value
		Negative (*n* = 43)	Positive (*n* = 27)	
Age, years (mean ± SD)		48.14 (11.77)	51.89 (12.12)	0.783
Sex	Female	16	10	1.000
	Male	27	17	
HBV infection	Absent	11	7	1.000
	Present	32	20	
Tumor number	Single	28	10	0.028 *
	Multiple	15	17	
Lymph Node Metastasis	Negative	21	17	0.326
	Positive	22	10	
Microvascular invasion	Absent	24	13	0.625
	Present	19	14	
Tumor differentiation	I–II	33	13	0.020 *
	III–IV	10	14	
TNM stage	I–II	32	12	0.021 *
	III	11	15	

HBV—hepatitis B virus; TNM—Tumor Node Metastasis. Tumor Differentiation: Well/Moderate (I–II) Poor/Undifferentiated (III–IV). * *p* < 0.05 was considered statistically significant.

## Data Availability

The raw data supporting the conclusions of this article will be made available by the authors, without undue reservation.

## Supplementary Materials

The following supporting information can be downloaded at: https://www.mdpi.com/article/10.3390/biomedicines14030631/s1. Supplementary Material for this article includes additional detailed methods, three supplementary figures, and one supplementary table. Figure S1: Expression profiles of the 35 DEGs in individual samples. Figure S2: Expression validation of hub genes in HC tissues. Figure S3: Original western blot images corresponding to Figures. Table S1: List of DEGs in HC tissues versus adjacent nontumorous tissues.

## Abbreviations

BP	biological process
CAMs	cell adhesion molecules
CC	cellular component
CNTN1	contactin-1
dDEGs	differentially expressed genes
DMEM	Dulbecco’s modified Eagle medium
ECM	extracellular matrix
EMT	epithelial–mesenchymal transition
FBS	fetal bovine serum
FDR	false discovery rate
GO	gene ontology
HC	hilar cholangiocarcinoma
IHC	immunohistochemical
KEGG	Kyoto Encyclopedia of Genes and Genomes
LV	lentiviral vector
MF	molecular function
PBS	phosphate-buffered saline
PI3K	phosphoinositide 3-kinase
PNI	perineural invasion
PPI	protein–protein interaction
RNA-seq	RNA sequencing
RT-qPCR	real-time quantitative polymerase chain reaction
SDS	sodium dodecyl sulfate
STR	short tandem repeat
