# Supplementary material for: Integrated Transcriptomic Analysis and Functional Validation Identify CNTN1 as a Novel Metastatic Driver in Hilar Cholangiocarcinoma
Source: Biomedicines. 2026 Mar 11;14(3):631. doi: 10.3390/biomedicines14030631 (PMC13023897; doi:10.3390/biomedicines14030631)
Supplement: Supplementary file 1 [file biomedicines-14-00631-s001.zip › biomedicines-4176295-supplementary.pdf]

Supplementary Materials

Integrated Transcriptomic Analysis and Functional Validation Identify CNTN1 as a Novel Metastatic Driver in Hilar Cholangiocarcinoma

Xiangming Ding [1,†], Chiyu Cai [2,†], Yuanxiang Lu [3,†], Zipeng Wang [3], Junjing Hou [3], Yushu Xue [1], Luyun Zhang [1], Meng Xie [1,*] and Dongxiao Li [1,*]

[1] Department of Gastroenterology, Henan Provincial People's Hospital, People's Hospital of Zhengzhou University, Zhengzhou 45003, China; dingxiangming@zzu.edu.cn (X.D.); xueyushu@gs.zzu.edu.cn (Y.X.); zhangluyun@gs.zzu.edu.cn (L.Z.)

[2] Department of Hepatobiliary Surgery, Henan Provincial People's Hospital, People's Hospital of Zhengzhou University, Zhengzhou 45003, China; chiyu1101@gs.zzu.edu.cn

[3] Department of Breast Surgery, Henan Provincial People's Hospital, People's Hospital of Zhengzhou University, Zhengzhou 45003, China; d202181970@hust.edu.cn (Y.L.); wzpzzu@gs.zzu.edu.cn (Z.W.); junjing151729@gs.zzu.edu.cn (J.H.)

* Correspondence: xiemeng@zzu.edu.cn (M.X.); lidongxiao@zzu.edu.cn (D.L.)

† These authors contributed equally to this work.

Materials and Methods

Identification of differentially expressed genes (DEGs)

DEGs between HC and non-HC tissues from the generated data (RNA-seq dataset) were screened using R scripts and Bayes tests were used to evaluate statistical significance.

Gene Ontology (GO) and Kyoto Encyclopedia of Genes and Genomes (KEGG) enrichment analyses

To elucidate the biological functions of the identified DEGs, Gene Ontology (GO) enrichment analysis was performed, categorizing genes into biological process (BP), cellular component (CC), and molecular function (MF) terms. KEGG pathway analysis was conducted to identify significantly enriched pathways associated with the DEGs. A hypergeometric test with a significance threshold of $P < 0.05$ was applied for both analyses.

Protein-Protein Interaction (PPI) Network Analysis and Hub Gene Identification

PPI network was constructed for the preliminary DEGs using the STRING database (version 11.0; https://string-db.org/) with a combined interaction score > 0.4 set as the cutoff. The network was visualized and analyzed using Cytoscape software (version 3.7.1). Significant functional modules within the network were identified using the MCODE plugin (version 1.5). Key hub genes were subsequently determined based on the network topology.

Western blotting

Protein extraction and western blotting were performed as previously described [1, 2]. Briefly, total proteins were separated by SDS-PAGE, transferred to PVDF membranes, and probed with specific primary antibodies overnight at 4°C. After incubation with appropriate HRP-conjugated secondary antibodies, protein bands were visualized using enhanced chemiluminescence (ECL) reagent.

Tissue microarray analysis and immunohistochemistry (IHC)

Tissue microarrays (TMAs) containing HC and adjacent non-tumorous tissues were constructed as previously described [1, 2]. For IHC, 4-μm-thick paraffin sections were deparaffinized, rehydrated, and subjected to antigen retrieval. Endogenous peroxidase activity was blocked with 3% H_2O_2. Sections were incubated with primary antibodies overnight at 4°C, followed by incubation with a horseradish peroxidase (HRP)-conjugated secondary antibody for 30 minutes at room temperature. Signal was developed using diaminobenzidine (DAB), and nuclei were counterstained with hematoxylin.

IHC staining was evaluated independently by two pathologists blinded to the clinical data. A semi-quantitative scoring system was applied based on both the percentage of positive cells (0: 0%; 1: 1–25%; 2: 26–50%; 3: 51–75%; 4: 76–100%) and staining intensity (0: negative; 1: weak; 2: moderate; 3: strong). The final immunoreactivity score (range 0–12) was calculated by multiplying the two scores. A score ≥ 4 was defined as positive expression.

References

1. Li D, Ding X, Xie M, Huang Z, Han P, Tian D, Xia L. CAMSAP2-mediated noncentrosomal microtubule acetylation drives hepatocellular carcinoma metastasis. Theranostics. 2020 Feb 19;10(8):3749-3766.
2. Xie M, Lin Z, Ji X, Luo X, Zhang Z, Sun M, Chen X, Zhang B, Liang H, Liu D, Feng Y, Wang Y, Li Y, Liu B, Huang W, Xia L. FGF19/FGFR4-mediated elevation of ETV4 facilitates hepatocellular carcinoma metastasis by upregulating PD-L1 and CCL2. J Hepatol. 2023 Jul;79(1):109-125.

Supplementary Figures and Table legends

Supplementary Figure S1. Expression profiles of the 35 DEGs in individual samples. Heatmap depicting the expression patterns of 35 DEGs across 3 individual hilar cholangiocarcinoma (Tumor) tissues and 3 paired adjacent non-tumorous tissues. The color scale represents Z-score normalized expression values across rows.

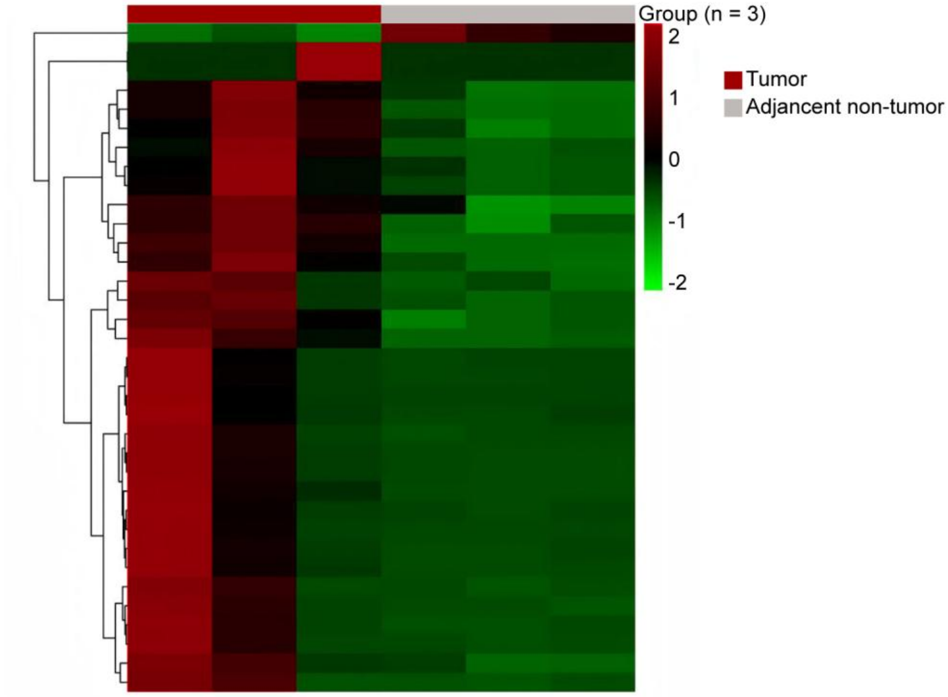

Supplementary Figure S2. Expression validation of hub genes in HC tissues. RT-qPCR analysis of the mRNA expression levels of the six identified hub genes (CNTN1, NCAM1, PLP1, GPM6B, SLC1A3, and PMP2) in 70 paired primary HC and adjacent non-neoplastic tissues. All reactions were performed in triplicate (n=3 technical replicates) for each sample.

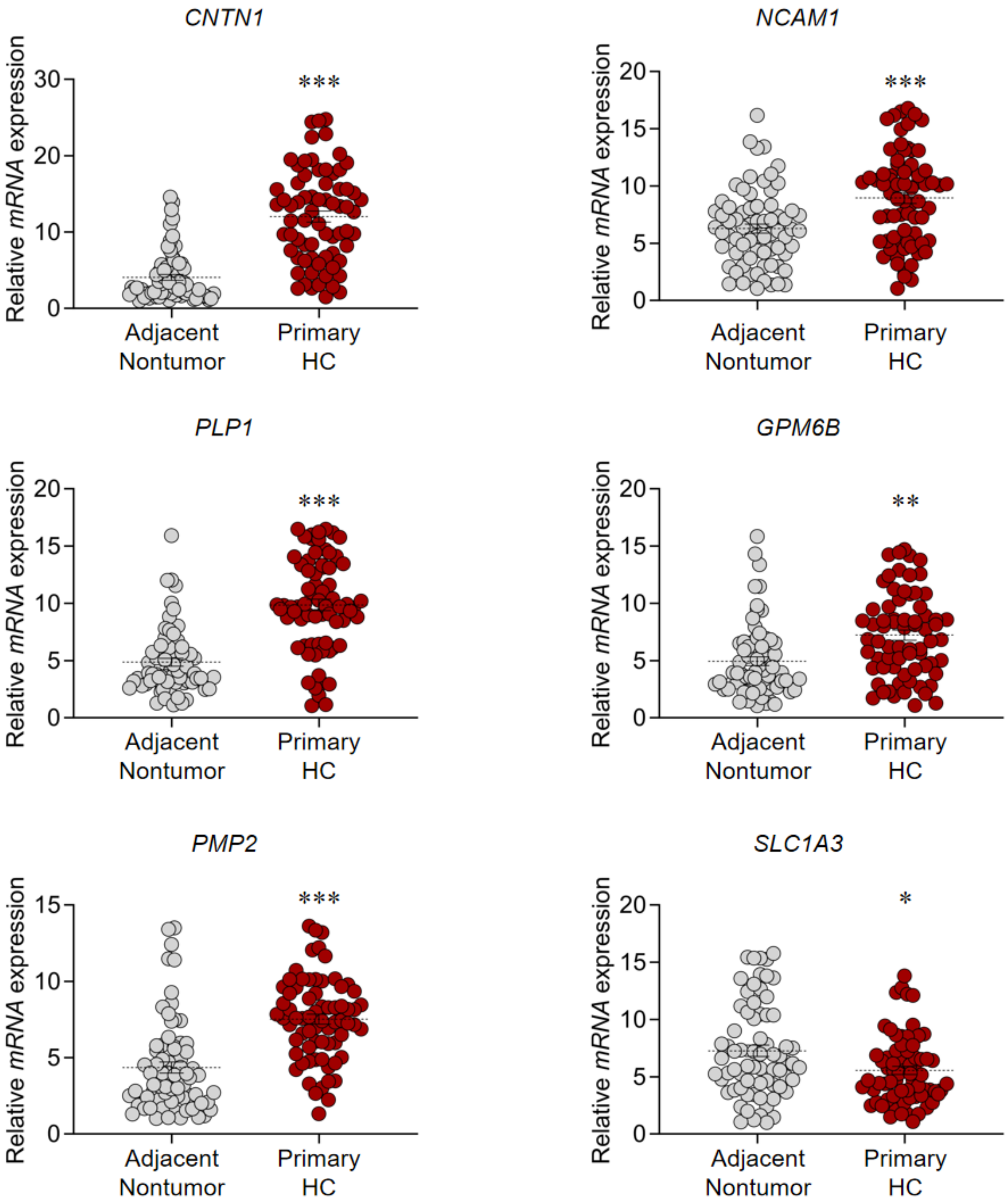

Supplementary Figure S3. Original western blot images corresponding to Figures.

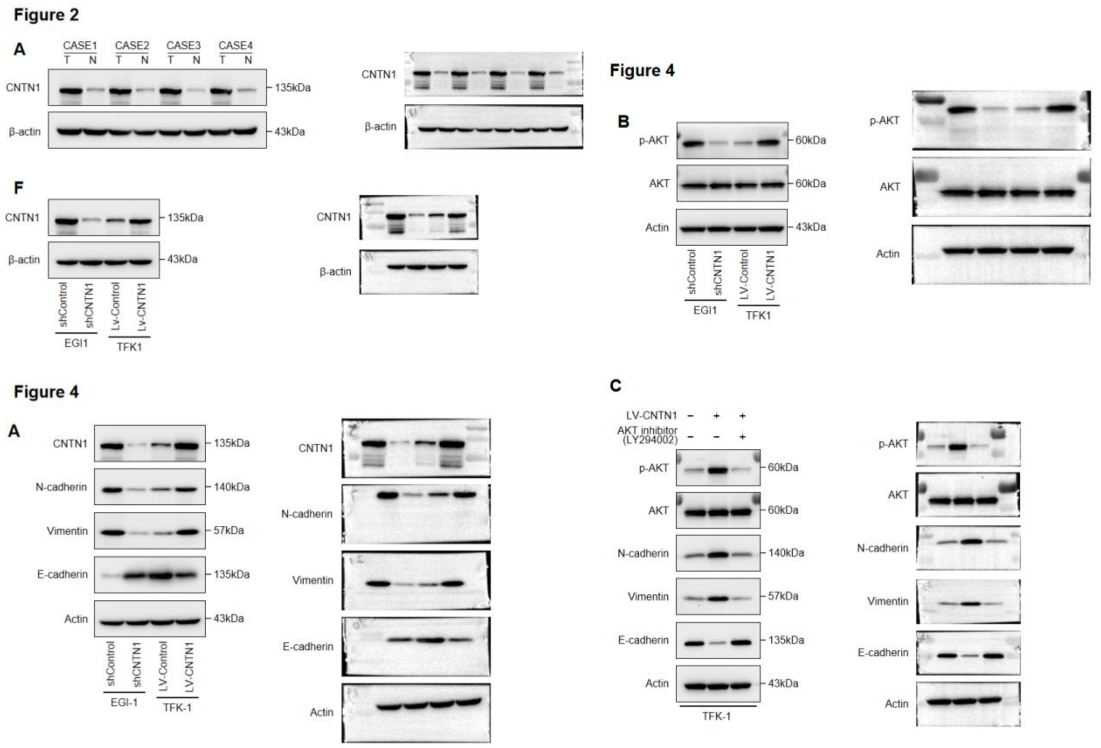

Supplementary Table S1. List of DEGs in HC tissues versus adjacent nontumorous tissues

Gene Symbol	Type	Normal expression	Tumor expression	log^2 (T/N)	*P* value (T/N)
CELA3B	mRNA	0.0042	0.733	7.4484	9.25E-09
CELA3A	mRNA	0.0099	1.59	7.3366	2.34E-09
XKR4	mRNA	0	0.3567	7.0654	3.73E-05
CNTN1	mRNA	0.18	20.0262	6.7976	9.37E-07
WRB-SH3BGR	mRNA	0	0.5733	6.217	3.86E-05
GRIK3	mRNA	0.1067	4.5433	5.229	2.69E-06
PMP2	mRNA	0.2433	8.9067	5.1451	3.31E-07
RASGEF1C	mRNA	0.0967	2.5833	4.6939	4.94E-06
TTYH1	mRNA	0.34	8.8533	4.6147	5.54E-06
COL28A1	mRNA	0.34	8.52	4.5617	2.05E-06
SORCS1	mRNA	0.17	3.7167	4.4038	3.05E-06
ANGPTL7	mRNA	0.8133	18.1933	4.3855	6.01E-05
MPZ	mRNA	3.2367	74.9833	4.3724	4.83E-06
PLP1	mRNA	2.1767	42.2733	4.1129	6.10E-06
INSC	mRNA	0.1667	2.5233	3.8475	1.23E-05
GPM6B	mRNA	1.2733	20.1167	3.8275	3.48E-06
PRIMA1	mRNA	1.5567	24.82	3.7892	4.91E-05
GFRA3	mRNA	1.3833	18.3567	3.6448	2.99E-05
CMTM5	mRNA	0.5467	6.86	3.557	2.95E-05
SCN7A	mRNA	0.7867	7.93	3.2364	6.94E-05
DES	mRNA	25.7233	234.6	3.1944	8.41E-15
PNCK	mRNA	0.22	1.95	3.0501	2.56E-05
EMILIN3	mRNA	0.1433	1.0933	2.9784	2.50E-05
MYH11	mRNA	34.8067	271.44	2.943	1.76E-06
NCAM1	mRNA	1.07	9.5733	2.9333	2.33E-05
SBSPON	mRNA	1.6867	12.8433	2.8134	3.12E-05
PPP2R2B	mRNA	1.0733	6.65	2.5439	6.22E-05
ITGA8	mRNA	1.6667	9.3867	2.4844	8.38E-05
MYOCD	mRNA	0.58	2.97	2.3918	3.69E-05
DAB1	mRNA	0.9	3.1933	2.2919	1.02E-11
HP	mRNA	7.7333	33.1167	1.9644	6.62E-06
SYNM	mRNA	4.33	14.8467	1.7181	2.80E-07
MYLK	mRNA	26.1967	81.2067	1.6164	9.67E-07
NFASC	mRNA	4.5133	7.64	0.7246	6.74E-05
SLC1A3	mRNA	13.6367	8.6333	-0.6999	8.89E-06
